# The Responses to CGRP in the Territory of the Posterior Cerebral Artery in Migraine

**DOI:** 10.1155/2022/2686689

**Published:** 2022-06-26

**Authors:** Darja Visočnik, Marjan Zaletel, Matija Zupan, Bojana Žvan

**Affiliations:** Department of Neurology, University Medical Centre Ljubljana, Zaloška cesta 2, 1000 Ljubljana, Slovenia

## Abstract

Calcitonin gene-related peptide (CGRP) is important in trigeminovascular (TMV) sensitization with neurogenic inflammation which might be involved in CGRP-induced headache (CGRP-IH). Distribution of white matter lesions, migraine aura, and functional neuroimaging indicate that posterior circulation is especially exposed to TMV sensitization. The transcranial Doppler (TCD) is able to detect changes in the posterior cerebral artery (PCA) during CGRP stimulation. Thus, we studied CGRP-induced hemodynamic changes in PCA and frequency of CGRP-IH. Twenty healthy subjects and 20 patients with migraine participated in our study. TCD was used to monitor mean arterial velocity in posterior cerebral artery (v_m_PCA). Simultaneously, end-tidal carbon dioxide (Et-CO_2_), mean arterial pressure (MAP), and heart rate (HR) were measured. During the experiment, we monitored the frequency of CGRP-IH. We determined the values of v_m_PCA, Et-CO_2_, MAP, and HR and calculate the response of v_m_PCA, Et-CO2, MAP, and HR to CGRP. To test the differences and relationships, statistical methods were applied using SSPS. We found significant decrease in v_m_PCA in migraine and control groups and found the v_m_PCA response to be significantly lower in migraine (*p* = 0.018). Et-CO_2_ decreases in both groups, and it is significantly lower in migraine (*p* < 0.001). MAP is significantly higher in migraine (*p* = 0.001), while HR is not significantly higher in migraine (*p* = 0.570). CGRP-IH is significantly associated with v_m_PCA responses (*p* = 0.003) and migraine (*p* < 0.001). We concluded that hemodynamic changes in PCA are significantly related to CGRP-IH. The TMV sensitization might be pronounced in posterior circulation explaining clinical and morphologic issues in migraine.

## 1. Introduction

According to current opinion, migraine episodes are linked to TMV sensitization. Neurogenic inflammation represents biological substrate of the TMV sensitization. Neurogenic inflammation might be also related to white matter changes in the posterior cerebral circulation in patients with migraine with aura. These white matter changes are supposed to be associated with the neuronal activity [1]. CGRP is a potent vasodilator and an important molecule in the process of sensitization [2]. In addition, clinical effective CGRP monoclonal antibodies inhibit CGRP and underlying sensitization [3]. Posterior circulation seems to be particularly vulnerable to sensitization.

Until now, only a few studies on the response of anterior cerebral circulation to CGRP intravenous infusion have been published. The results of these studies on effects of CGRP infusion on the middle cerebral artery (MCA) were conflicting. In TCD study in healthy subjects, vasodilatation of MCA was found, but in TCD study in migraine patients, no noticeable changes of MCA diameter were observed [4, 5]. In the third MRA study, no differences in the MCA diameter were detected before and after CGRP infusion [6]. In our recent study in healthy subjects, we found hemodynamic responses of MCA and PCA to CGRP infusion [7] By our knowledge, so far this was the only study of PCA reactivity to CGRP infusion. Therefore, at present, there is no available data considering PCA response to CGRP in migraine patients. Multimodal TCD is a noninvasive method for evaluating cerebral circulation and has access to posterior cerebral territory through temporal acoustic window.

The TMV sensitization of posterior circulation could be considered an ongoing process which is clinically manifested as headache during migraine episodes. But it could be occurring even interictally. In migraine patients during interictal periods, the CGRP provocation could intensify the TMV sensitization and evoke CGRP-induced headaches (CGRP-IH) [8] In the present study, we investigated the hemodynamic and clinical effects of CGRP on PCA interictally in migraine patients. We predicted that hemodynamic response to CGRP PCA territory and occurrence of CGRP-IH are increased in patients with migraine. The article is at the time of writing available as a preprint [9]

## 2. Materials and Methods

In our study, we included twenty healthy subjects (9 females aged 37.0 ± 2.8 years, 11 males aged 41.8 ± 7.6 years) and 20 patients with migraine (15 females aged 41.9 ± 9.9 years, 5 males aged 38.2 ± 9.2 years). The number of the participants in the study was driven from previous studies [4, 5, 6]. We did not find significant differences in sex (*p* = 0.105) nor in age (*p* = 0.066) between the groups. The inclusion criteria for the group of healthy participants were negative family history for migraine and age 18 or more. The inclusion criteria for the migraine group were migraine with or without aura in accordance with the ICHD-3 criteria of the International Headache Society [10] and age 18 or more. The exclusion criteria for the healthy group and migraine group were the presence of cerebrovascular, renal, or liver diseases and uncontrolled endocrine diseases, uncontrolled hypertension, pregnancy, breastfeeding, abnormalities of somatic and neurological status, and hemodynamic important atherosclerotic process of carotid or vertebral arteries.

The participants were free of tobacco, coffee, tea, or any other food or beverages containing caffeine for at least 12 hours before the start of the measurements.

All participants were given written explanation regarding the experimental procedure. They all gave written consent to participate in the study. The study was approved by the National Medical Ethics Committee of the Republic of Slovenia.

Before the beginning of the experiment, color-coded duplex sonography of the carotid and vertebral arteries was performed using the standard procedure. The experiments took place at 9:00 am in a quiet and darkened room under constant conditions. During the experiment, participants were resting in the supine position. The experiment lasted 40 min including a 10 min baseline period, a 20 min period during which an intravenous infusion of human alpha CGRP 1.5 mcg/min (Calbiochem, Merck Biosciences, Darmstadt, Germany) was given, and a 10 min period after the application of CGRP. The CGRP dose of 1.5 mcg/min was chosen due to results of previous studies, which showed that it is safe and caused no profound hypotension. TCD sonography with a 2 MHz ultrasound probe was applied to measure v_m_PCA through the right transtemporal acoustic window. The signal of the PCA was defined according to the direction of the blood flow, the typical depth of the signal, and the response to closing eyes. A mechanical probe holder was used to ensure a constant probe position. During the entire experiment, MAP and heart rate (HR) were continuously measured using noninvasive plethysmography (Colin 7000, 12 Komaki City, Japan). Et-CO_2_ was measured in exhaled breath using a ventilation mask and an infrared capnometer (Capnograph, Model 9004, Smith Medical, USA).

TCD Multi-Dop X4 software (DWL, Sipplingen, Germany) was used to define and average integrals of 5-minute values for v_m_PCA, MAP, HR, and Et-CO_2_. We calculated an average integral of 5 min values using the following equation for v_m_PCA : v_m_PCA═∫*vdt*/(*t*_0_min–*t*_5_min). The same equation was used to calculate an average integral for the rest of the variables (MAP, HR, and Et-CO_2_).

For further statistical analysis, we defined data of 5 min intervals as measuring points *T*_0_, *T*_1_, *T*_2_, and *T*_3_. Measurement point *T*_0_ represented an interval during the baseline period before starting CGRP infusion (5-10 min of the experiment), *T*_1_ was a 5-minute interval in the first part of CGRP infusion (15-20 of the experiment), *T*_2_ represented a 5-minute interval in the last part of CGRP infusion (25-30 minutes of the experiment), and *T*_3_ was the 5-minute interval in the last part of the experiment after the end of CGRP infusion (35-40 min of the experiment).

In the next step, we calculated the responses for v_m_PCA, Et-CO_2_, HR, and MAP, respectively, as differences between measuring points. Response 1 represented the difference between measurement points *T*_1_ and *T*_0_, response 2 represented the difference between measurement points *T*_2_ and *T*_0_, and response 3 represented the difference between measurement points *T*_3_ and *T*_0_.

After that we performed statistical analysis of all three responses as one variable (v_m_PCA_tot_, Et-CO_2tot_, HR_tot_, and MAP_tot_). Our assumption was based on CGRP pharmacokinetic studies [11–12] which confirmed that CGRP was active in the time period of *T*_1_, *T*_2_, and *T*_3_ measurement points.

We determine CGRP-IH by using ICHD-3 criteria [10]. We did not calculate the frequency of immediate CGRP-IH and delayed CGRP-IH separately but together as one variable.

For statistical analysis, SPSS version 21 was used. The paired *t*-test and Student *t*-test were used to test the significance of differences between dependent and independent variables. Linear regression and logistic regressions were used to test the correlations between the variables. Normality of variability distribution was tested, and all variables had value of the Shapiro-Wilk test greater than 0.05. The results in the statistic tests were statistically significant if *p* < 0.005.

## 3. Results

**Figure 1:**
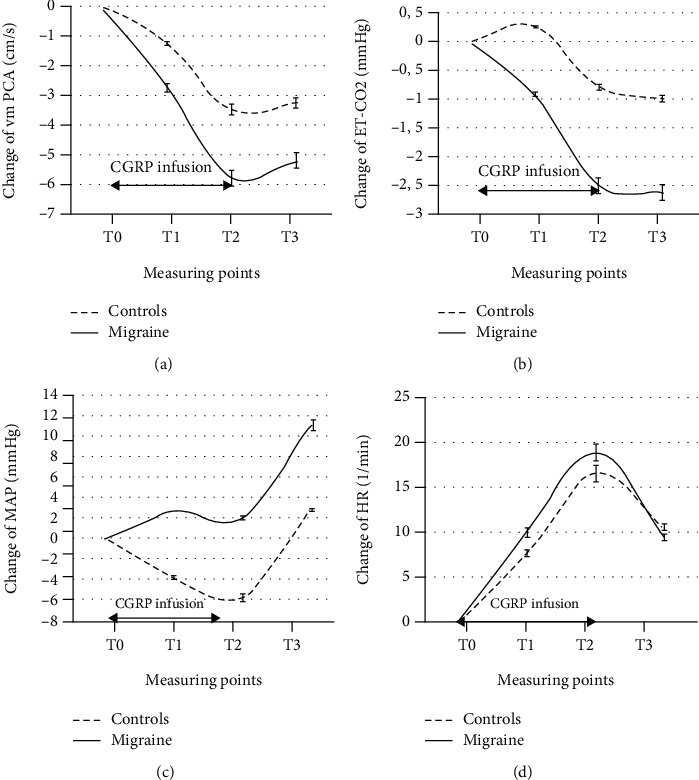
Changes of v_m_PCA, Et-CO_2_, MAP, and HR during and after CGRP infusion in the control and migraine groups. Legend: v_m_PCA: mean blood flow velocity in the posterior cerebral artery; Et-CO_2_: end-tidal CO_2_; MAP: mean arterial pressure; HR: hart rate.

The frequency of CGRP-IH was higher in the migraine group than in the control group. In the control group, we found 4 subjects (20%), and in the migraine group, there were 16 patients (80%) with CGRP-IH. The difference in frequency of CGRP-IH between the groups was significant (*p* < 0.001). Logistic regression showed a significant association between migraine and CGRP-IH (OR = 16.00; 95% CI 3.39-75.34; *p* < 0.001). In Figure 1, we present the time course of v_m_PCA, Et-CO_2_, MAP, and HR changes using *T*_0_ as the baseline point and *T*_1_, *T*_2_, and *T*_3_ as points obtained during and after CGRP infusion when CGRP was acting as a stimulus for cerebral and systemic circulation for control and migraine groups.

Statistically significant differences between measuring points for all variables are shown in Table 1 for the control group and migraine group.

**Figure 2:**
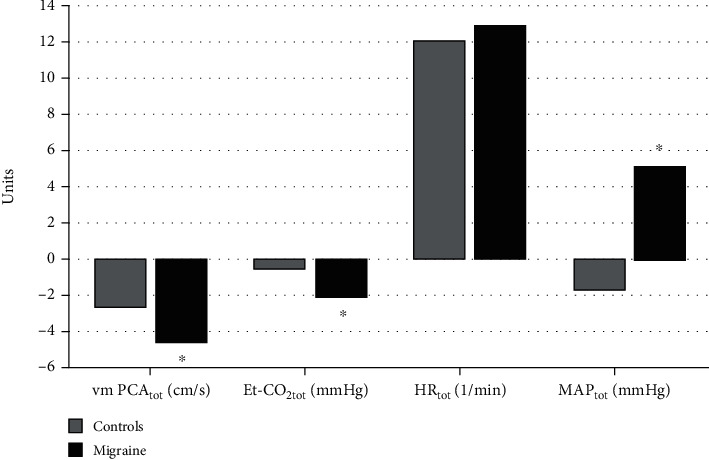
Differences between the control and migraine groups in v_m_PCA_tot_, Et-CO_2tot_, MAP_tot_, and HR_tot_ responses. Legend: vmPCA_tot_: mean blood flow velocity in the posterior cerebral artery total response; Et-CO_2tot_: end-tidal CO_2_ total response; MAP_tot_: mean arterial pressure total response; HR_tot_: heart rate total response.

In the next step, we analyzed the differences in responses to CGRP between control and migraine groups using *t*-test. All three responses (responses 1, 2, and 3) of each parameter separately (v_m_PCA, Et-CO_2_, MAP, and HR) were combined into a single variable (Figure 2). We found that v_m_PCA_tot_ (*p* = 0.018) and Et-CO_2tot_ (*p* < 0.001) in the migraine group are significantly greater compared to those in the control group. MAP_tot_ response was significantly higher in migraineurs compared to controls (*p* = 0.001), while HR_tot_ response did not differ significantly between the groups (*p* = 0.570).

In the control and migraine group, CGRP-IH is associated with v_m_PCA_tot_ responses (OR = 1.19; 95% CI 1.06-1.33; *p* = 0.003) and Et-CO_2tot_ responses (OR = 1.31; 95% CI 1.08-1.59; *p* = 0.006) but not with MAP_tot_ responses (OR = 0.97; 95% CI 0.94-1.01; *p* = 0.221) and HR_tot_ responses (OR = 1.00; 95% CI 0.95-1.06; *p* = 0.784).

We also found significant linear relationships between v_m_PCA_tot_ and Et-CO_2tot_ responses (*B* = 0.17; beta = 0.34; *p* < 0.001) as well as between v_m_PCA_tot_ and MAP_tot_ responses (*B* = 0.53; beta = 0.22; *p* = 0.017) but not between v_m_PCA_tot_ and HR_tot_ (*B* = −0.15; beta = −0.096; *p* = 0.30).

We performed logistic regression analysis between migraine and physiologic variables. We found significant associations between migraine and v_m_PCA_tot_ (OR = 1.12; 95% CI 1.01-1.23; *p* = 0.025), Et-CO_2tot_ (OR = 1.45; 95% CI 1.17-1.80; *p* = 0.001), and MAP_tot_ (OR = 0.98; 95% CI 0.90-0.97; *p* = 0.001) responses but not between migraine and HR_tot_ response (OR = 0.98; 95% CI 0.93-1.03; *p* = 0.567).

## 4. Discussion

The main finding of our study is higher v_m_PCA_tot_ response to CGRP in the migraine group than in the control group. This finding is consistent with our assumption of TMV sensitization. According to the current knowledge, CGRP evokes vasodilatation of the proximal cerebral segment, which tends to increase cerebral blood flow (CBF). Namely, cerebral circulation differs from systemic one in resistance distribution between proximal and distal segments. It is well accepted that the proximal segment contributes about 50% of cerebral vascular resistance [13] Therefore, proximal segments of cerebral circulation can importantly influence CBF which is strictly regulated. Thus, a mechanism which tends to normalize CBF must exist. The studies on CGRP influence on cerebral circulation noticed lowering of Et-CO_2_ during CGRP activity [5]. In the present study, we detected Et-CO_2_ decrease in controls and even more pronounced Et-CO_2_ decrease in the migraine group. We supposed that this phenomenon is not an incidental one, but it could have a regulatory role in providing constant CBF. It is well known that pCO_2_ has a strong effect on CBF and is supposed to act on the distal segment of cerebral arteries modulating cerebral resistance [13]. Therefore, lowering of Et-CO_2_ during CGRP stimulation could be considered a protective effect against undesired effects of increased CBF. Basically, it could represent a negative feedback loop for CBF regulation. Indeed, we noticed that Et-CO_2_ is directly proportional to v_m_PCA decrease during CGRP infusion. This could be explained by vasodilation of PCA and Et-CO_2_ compensatory effect.

The physiologic effects and mechanism of exogenous CGRP action are still a challenging issue. We suppose that CGRP acts on cerebrovascular smooth muscle cells to increase intracellular cAMP, decrease intracellular calcium ions, and cause vasorelaxation. However, the CGRP has no effect on the cerebrovascular endothelium [14]. In the previous experiments, CGRP was effective in relaxing the artery only when applied to the abluminal surface of the vessel. However, CGRP receptor antagonists and anti-CGRP antibodies blocked CGRP-mediated dilation when they are applied to the lumen of the arteries [15]. Therefore, intravenous CGRP should not produce direct vasodilatation of PCA. It is well known that trigeminal ganglion is lacking a hematoencephalic barrier and is readily accessible to exogenic CGRP and possesses CGRP receptors [16]. The described facts are enabling exogenous CGRP to sensitize the TMV complex through trigeminal ganglion neurons. In turn, the TMV sensitization caused vasodilatation of PCA. Our results suggest that the PCA dilatation relates to the degree of TMV sensitization.

On general, migraine headache represents clinical correlate of TMV sensitization. According to our previous deliberations, exogenic CGRP evokes TMV sensitization. In addition, sensitization of the TMV complex could be considered an ongoing process with different levels of activity. Therefore, exogenous CGRP intensifies the TMV activity and could produce headache when the pain threshold is exceeded. We noted high frequency of CGRP-IH in the migraine group which is in accordance with the previous studies [17]. However, not all migraineurs suffered from CGRP-IH. This is probably due to lower TMV sensitization activity. Interestingly, we noticed CGRP-IH also in the control, nonmigraine group. We could explain the phenomena by relatively high enough ongoing TMV sensitization activity to evoke CGRP-IH in those nonmigraineurs. Nevertheless, we found the association between migraine and CGRP-IH with high odds ratio which supports our assumptions.

In our study, we found the relationship between CGRP-IH and v_m_PCA_tot_ response supporting our premises on sensitization and vascular inflammation in posterior circulation during migraine headache. In addition, we found relationship between CGRP-IH and Et-CO_2tot_ response which supports our assumption that partial artery pressure of carbon dioxide might be a regulatory mechanism of CBF during CGRP stimulation. The association between migraine and occipital lobe activity is a longstanding matter of debate. Studies have shown an increased white matter lesion load in posterior circulation [18]. The pathogenesis of these lesions is still unclear, but neurogenic inflammation during sensitization could be one of the putative causes which directly or indirectly produce damage of white matter. The neuronal activity in the posterior circulation during a migraine episode was also recognized by functional neuroimaging [19]. The importance of posterior circulation in migraine pathogenesis was supported by previous studies [20]. This could explain why posterior circulation is specifically affected during sensitization.

In the present study, we followed changes in systemic circulation monitoring HR and MAP during and after CGRP infusion. In the control group, we observed a significant decrease in MAP during CGRP infusion and normalization after it. In the migraine group, we did not observe significant changes during CGRP infusion but marked increase in MAP after it. We found statistically significant differences in MAP_tot_ responses between the groups. In the control and migraine group, HR expectedly increases during CGRP infusion and decreases after it. According to studies, intravenous administration of CGRP leads to positive chronotropic and inotropic effects associated with reduction in blood pressure and rise of plasma noradrenalin and adrenalin levels [21]. The subsequent sympathetic activation with release of catecholamines displays the indirect effects of CGRP which we clearly observed in healthy subjects. The reaction of the migraine group is unexpected. It is well known that during normal physiologic conditions, the plasma concentration of CGRP is low. In general, the level of CGRP in the systemic circulation in humans is limited to the picomolar range [15]. In this range, CGRP is not thought to have an effect on the systemic circulation. However, as mentioned, the plasma concentration of CGRP is elevated during migraine attacks in association with neurogenic inflammation [22], and due to TMV sensitization, it drains CGRP from the brain. Indeed, CGRP levels in peripheral plasma, saliva, and cerebrospinal fluid are increased during interictal, headache-free periods in patients with chronic migraine [23]. Thus, exposure of systemic circulation to CGRP is more pronounced in migraineurs. According to our results, it seems that systemic circulation of migraine patients is not sensitive to CGRP effects during the infusion period. Relative insensitivity to CGRP in migraine could be attributed to higher catecholamine activity. The intensive upstroke of MAP in migraine patients after CGRP infusion could be a consequence of higher noradrenergic systemic hyperactivity. Indeed, studies report increased sympathetic activity [24] and also parasympathetic hypofunction [25]. Recent study indicates a slight difference in the balance between sympathetic and parasympathetic activity with a net increase in sympathetic tone [26].

An underpowered statistic is a limitation of our study, but it still provides valuable information on CGRP effects and may represent a basis for future studies. Our study may serve as a substrate for estimation of sample size to ensure an appropriate study power in similar future studies. Furthermore, the study lacks data on CGRP plasma concentration, and it did not explore nocebo contribution in the appearance of CGRP-IH.

## 5. Conclusions

In conclusion, our study provides evidence for TMV sensitization of the posterior circulation in migraine. It seems that TMV sensitization is active even in nonmigraineurs and could be intensified and clinically manifested with exogenous CGRP. Posterior circulation might be more exposed to TMV sensitization and neurogenic inflammation in comparison to other parts of cerebral circulation. The systemic circulation shows slight differences between migraine and control groups. This is probably due to long-term effects of CGRP released into systemic circulation during migraine episodes and its long-term effects on the autonomic nervous system.

## Figures and Tables

**Table 1:** Statistically significant differences between measuring points in the control and migraine groups.

	*T*_1_-*T*_0_	*T*_2_-*T*_0_	*T*_3_-*T*_0_
Control group			
v_m_PCA	*p* = 0.003	*p* < 0.001	*p* = 0.002
Et-CO_2_	*p* = 0.376	*p* = 0.023	*p* = 0.066
MAP	*p* = 0.062	*p* = 0.027	*p* = 0.119
HR	*p* < 0.001	*p* < 0.001	*p* < 0.001
Migraine group			
v_m_PCA	*p* = 0.015	*p* < 0.001	*p* < 0.001
Et-CO_2_	*p* = 0.018	*p* < 0.001	*p* = 0.001
MAP	*p* = 0.226	*p* = 0.557	*p* = 0.001
HR	*p* < 0.001	*p* < 0.001	*p* < 0.001

Legend: v_m_PCA: mean blood flow velocity in the posterior cerebral artery; Et-CO_2_: end-tidal CO_2_; MAP: mean arterial pressure; HR: hart rate.

## Data Availability

The data used and analyzed during current study are available from the corresponding author on reasonable request.
